# Thrombin‐mediated activation of Akt signaling contributes to pulmonary vascular remodeling in pulmonary hypertension

**DOI:** 10.1002/phy2.190

**Published:** 2013-12-29

**Authors:** Aiko Ogawa, Amy L. Firth, Sanae Ariyasu, Ichiro Yamadori, Hiromi Matsubara, Shanshan Song, Dustin R. Fraidenburg, Jason X.‐J. Yuan

**Affiliations:** 1 Department of Clinical Science, National Hospital Organization Okayama Medical CenterTamasu, Kita‐kuOkayama, Japan; 2 The Salk Institute of Biological Studies, La Jolla, California; 3 Clinical Pathology, National Hospital Organization Okayama Medical Center, TamasuKita‐kuOkayama, Japan; 4 Department of Medicine, Division of Pulmonary, Critical Care, Sleep and Allergy Medicine, University of Illinois at Chicago, Chicago, Illinois; 5 Center for Cardiovascular Research, University of Illinois at Chicago, Chicago, Illinois; 6 Department of Pharmacology, University of Illinois at Chicago, Chicago, Illinois

**Keywords:** Akt signaling, chronic thromboembolic pulmonary hypertension, pulmonary hypertension, store‐operated calcium entry, thrombin

## Abstract

Chronic thromboembolic pulmonary hypertension (CTEPH) has been increasingly recognized as a common source of elevated pulmonary vascular resistance and pulmonary hypertension. It is clear that development of pulmonary thromboemboli is the inciting event for this process, yet it remains unclear why some patients have persistent pulmonary artery occlusion leading to distal pulmonary vascular remodeling and CTEPH. Thrombin, a serine protease, is an integral part of the common coagulation cascade, yet thrombin also has direct cellular effects through interaction with the family of PAR membrane receptors. This study is designed to determine the effects of thrombin on Akt signaling in pulmonary artery smooth muscle cells (PASMC) from normal humans and pulmonary hypertension patients. Thrombin treatment of PASMC resulted in a transient increase in Akt phosphorylation and had similar effects on the downstream targets of the Akt/mTOR pathway. Ca^2+^ is shown to be required for Akt phosphorylation as well as serum starvation, a distinct effect compared to platelet‐derived growth factor. Thrombin treatment was associated with a rise in intracellular [Ca^2+^] and enhanced store‐operated calcium entry (SOCE). These effects lead to enhanced proliferation, which is more dramatic in both IPAH and CTEPH PASMC. Enhanced proliferation is also shown to be attenuated by inhibition of Akt/mTOR in CTEPH PASMC. Thrombin has direct effects on PASMC increasing intracellular [Ca^2+^] and PASMC proliferation, an effect attributed to Akt phosphorylation. The current results implicate the effects of thrombin in the pathogenesis of idiopathic pulmonary arterial hypertension (IPAH) and CTEPH, which may potentially be a novel therapeutic target.

## Abstract (precis)

Thrombin is known to play an important role in thrombotic events including pulmonary embolism. In this manuscript, we show a direct effect of thrombin on pulmonary artery smooth muscle cells in both normal and diseased states through Akt signaling, which leads to increased store‐operated calcium entry and cellular proliferation. These direct effects of thrombin may play a role in the development and progression of chronic thromboembolic pulmonary hypertension.

## Introduction

Chronic thromboembolic pulmonary hypertension (CTEPH) results from migration and persistence of thromboemboli, usually from the deep leg veins, into the proximal pulmonary arterial vasculature (Dartevelle et al. 2004). Venous thromboembolism represents a common disease that results when the circumstances of Virchow's triad coalesce: stasis of blood flow, endothelial damage, and hypercoaguability. Imaging modalities including ventilation–perfusion scintigraphy identify the proximal perfusion defect and pulmonary hypertension is confirmed by right heart catheterization showing increased pulmonary vascular resistance (PVR) and mean pulmonary artery pressure (PAP) (Auger et al. 2012). Thrombi lodged in the proximal pulmonary arteries lead to increased pulmonary arterial pressure through both direct consequence of obstructed blood flow due to the thrombus as well as indirect effects of pulmonary vascular remodeling in distal pulmonary arteries (Auger et al. 1992; Moser and Bloor 1993; Burrowes et al. 2011). The incidence of CTEPH occurring after pulmonary embolism has been shown to be around 3% (Pengo et al. 2004; Ozsu and Cinarka 2013), and though risk factors have been identified, questions remain as to the molecular and biochemical factors that predispose to retained thrombi and vascular remodeling rather than spontaneous resolution (Sacks et al. 2006).

Our recent study examining isolated cells from endarterectomized human tissue has revealed a substantial presence of both endothelial and smooth muscle progenitor cells (Yao et al. 2009; Firth et al. 2010). As a common component of both the intrinsic and extrinsic clotting cascade, thrombin plays an important role in thrombus formation, cleaving fibrinogen to insoluble fibrin. Thus, increased levels of circulating thrombin are expected in association with thrombotic disease, and its cellular effects may be implicated in the development and/or progression of CTEPH. Wu and colleagues have shown that thrombin can stimulate a procoagulant endothelial phenotype by modulating T‐type calcium channels in pulmonary artery endothelial cells (PAEC) (Wu et al. 2003). Our previous study has shown that thrombin treatment for 72 h in PAEC results in increased [Ca^2+^]_cyt_.(Firth et al. 2009) This increased [Ca^2+^]_cyt_ is thought to result in endothelial cell dysfunction, a proposed mechanism for CTEPH development (Sakao et al. 2011). These cellular effects of thrombin are mediated by protease‐activated receptors (PARs), in which the N‐terminus of the receptor is cleaved by thrombin. This cleaved receptor can then act as a ligand that has been shown to mediate cell contraction, proliferation, and hypertrophy (Hauck et al. 1999; Pawlinski et al. 2007). PAR‐2 has recently been shown to be increased in both human IPAH and experimental animal models of pulmonary hypertension and its inhibition was able to reverse experimental hypoxia‐induced pulmonary hypertension (Kwapiszewska et al. 2012). Thrombin receptor activation has indeed been shown to induce a number of downstream signaling events, including stimulation of endogenous platelet‐derived growth factor (PDGF)‐A production (Wu and Aird 2005). We have shown in a previous study that a high deposition of PDGF is observed in distal arteries of patients with CTEPH and increased expression of PDGF receptor (PDGFR) in cells isolated from endarterectomized tissues of CTEPH patients (Ogawa et al. 2009).

This study is designed to detect the effects of thrombin on cellular mechanisms that could potentially lead to the pulmonary vascular remodeling observed in CTEPH and IPAH. We are able to demonstrate that thrombin increases cell proliferation through enhanced, calcium‐dependent Akt/mTOR signaling.

## Methods

### Chemicals, Antibodies, and Materials

PDGF‐BB, EGTA, and cyclopiazonic acid (CPA) were purchased from Sigma‐Aldrich. Human *α*‐thrombin was purchased from Enzyme Research Laboratories. Rapamycin and Akt inhibitor (VIII) were purchased from Calbiochem. Antibodies to mTOR, phospho‐mTOR (Ser2448), p70S6K, phospho‐p70S6K (Thr389), Akt, phospho‐Akt (Ser473), 4EBP1, and phospho‐4EBP1 (Ser65), eIF4E, phospho‐eIF4E (Ser209), ERK, *p‐*ERK1/2 (Thr202/Tyr204) were purchased from Cell Signaling Technology. Antibody for human smooth muscle actin was purchased from Dako. Antibody to GAPDH and HRP‐conjugated donkey anti‐mouse IgG antibody were purchased from Millipore. Antibodies to PAR1 and HRP‐conjugated anti‐rabbit IgG antibody were from Santa Cruz Biotechnology.

### Cell preparation and culture

Endarterectomized tissue was obtained from patients with CTEPH during pulmonary endarterectomy. All experiments were carried out after the approval of our protocol by the Institutional Review Board of University of California, San Diego (La Jolla, CA). Written informed consent was obtained from all patients before the procedure. Cell isolation was performed as previously reported (Ogawa et al. 2009). Human PASMC and PAEC from normal subjects were purchased from Lonza. PASMC and PAEC were cultured in smooth muscle growth media (Lonza) and endothelial growth medium (Lonza), respectively. When serum starvation is needed, 0.1% fetal bovine serum (FBS) was added to basal medium without adding other growth factors. All the cells were incubated in a humidified 5% CO_2_ atmosphere at 37°C. After reaching confluence, the cells were subcultured by trypsinization with 0.05% trypsin‐EDTA (Lonza).

### Immunohistochemistry

Lung tissue was obtained by autopsy at Okayama Medical Center. Immunohistochemical studies were performed using formalin‐fixed paraffin‐embedded sections. Histological sections were stained with Elastica‐Masson's trichrome stain. Immunostaining for PAR1 and smooth muscle actin was performed with an automated stainer, BenchMark XT Instrument (Ventana Medical Systems) using the iView staining kit (Ventana), according to the manufacturer's protocol.

### Thymidine Uptake Assay

^3^H‐thymidine incorporation assay was performed to assess DNA synthesis and proliferation of cells. Cells were seeded in 12‐well plates and treated for each experiment. Twenty‐four hours before treatment is finished, 1 *μ*Ci [^3^H]‐thymidine was added to each conditioned media. After 1 day, cells were washed with cold PBS once and washed twice with cold 7.5% trichloracetic acid, and then lysed with 0.5 M NaOH. The radioactivity was measured in a liquid scintillation counter. The data were obtained as counts per minutes.

### Western blot analysis

The cells were washed with ice‐cold PBS, suspended into lysis buffer (1% Nonidet P‐40, 0.5% sodium deoxycholate, 0.1% sodium dodecyl sulfate, 100 mg/ml phenylmethylsulfonyl fluoride, phosphatase inhibitors, and protease inhibitors), and incubated for 30 min on ice. The cell lysates were then sonicated, centrifuged at 12,000 rpm for 10 min, and the supernatant was collected. Samples were applied on SDS‐PAGE (4–20%) and proteins were transferred onto nitrocellulose membranes by electroblotting. Membranes were blocked in 5% nonfat milk, incubated overnight at 4°C with primary antibodies and then with secondary antibody. Blots were developed using the SuperSignal West Pico Chemiluminescent Substrate (Pierce Biotechnology).

### Measurement of [Ca^2+^]_cyt_

Cells were plated on 25‐mm coverslips and placed in a recording chamber on the stage of an inverted Nikon Eclipse/TE 200 microscope with the TE‐FM epifluorescence attachment. Cytoplasmic Ca^2+^ concentration ([Ca^2+^]_cyt_) was measured in each cell using the membrane‐permeable Ca^2+^‐sensitive fluorescent indicator, Fura‐2‐AM (Invitrogen). The cells were incubated at room temperature for 30 min in modified Krebs solution (MKS) containing 4 *μ*mol/L Fura‐2‐AM. The loaded cells were then washed with MKS for 30 min to remove excess extracellular dye and allow intracellular esterases to cleave cytosolic Fura‐2‐AM into active Fura‐2. Fura‐2 fluorescence was observed as 510‐nm‐wavelength light emission with excitation wavelengths of 340 and 380 nm by using the digital fluorescence imaging system from Intracellular Imaging. In all experiments, multiple cells were imaged in a single field, and one arbitrarily chosen peripheral cytosolic area from each cell was spatially averaged. [Ca^2+^]_cyt_ was expressed as Fura‐2 fluorescence emission ratio excited at 340 and 380nm (F_340_/F_380_).

### Statistical analysis

The data are expressed as means ± SEM. Differences between groups were examined for statistical significance using Student's *t*‐test or one‐way analysis of variance followed by Tukey's test. Differences were considered to be statistically significant when *P *<**0.05.

## Results

### Thrombin treatment is associated with transient Akt and mTOR phosphorylation in PASMC, but not in PAEC

**Figure 1.**
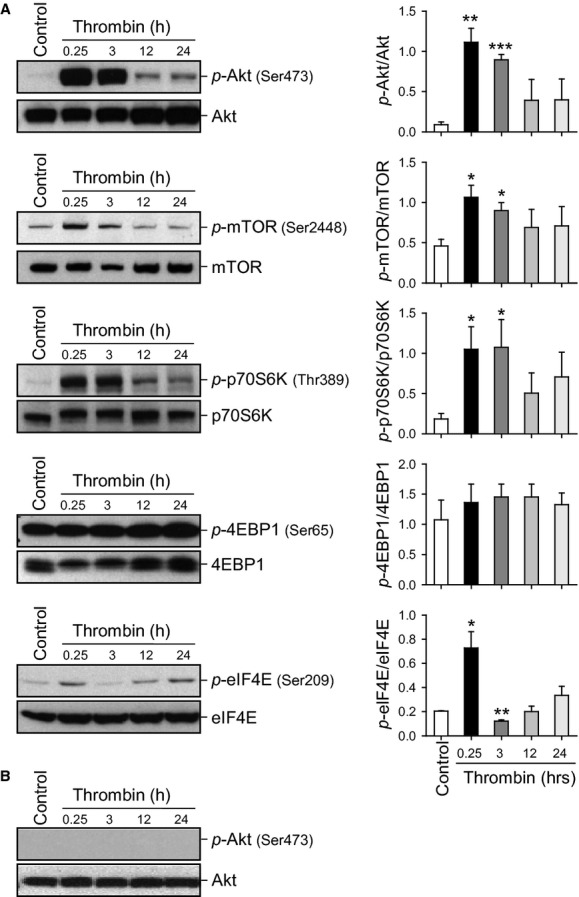
Time course of thrombin‐induced Akt/mTOR pathway phosphorylation in PASMC and PAEC. (A) Representative Western blots with bar graphs showing time‐dependent changes in phosphorylated and total proteins of Akt/mTOR pathway induced by thrombin stimulation in normal PASMC. Protein expression was assessed at 0.25, 3, 12, and 24 h after thrombin treatment. Summary data (mean ± SE, *n* = 3) were quantitated and compared at each time point with the control being without thrombin treatment. *P <**0.05 and **P <**0.01 versus Control (Cont) bars. (B) Representative Western blots showing no phosphorylation of Akt induced by thrombin (100 nmol/L). Blots were representative of 3 independent sets of experiments.

Thrombin (100 nmol/L)‐induced marked phosphorylation of the Akt/mTOR pathway in normal PASMC (Fig. 1). Cells were serum‐starved for 48 h and the effect of thrombin was assessed over a 24‐h time period, representative western blots are shown along with summarized data (Fig. 1A). Thrombin transiently increased phosphorylation of Akt and subsequently caused marked increase in phosphorylated mTOR, p70S6K, and eIF4E (12.6‐, 2.3‐, 5.7‐, 3.6‐fold increase in 15 min, *P* < 0.01, 0.05, 0.05, 0.01, respectively) in PASMC. Interestingly, in PAEC, there is no concurrent increase in the phosphorylation of Akt in response to stimulation by thrombin when studied over the same time period as PASMC (Fig. 1B).

### Thrombin‐mediated Akt activation/phosphorylation in PASMC is Ca^2+^ dependent

**Figure 2.**
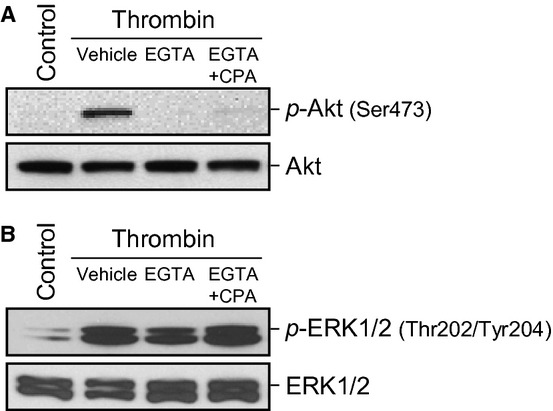
Akt activation induced by thrombin is partly dependent of extracellular and intracellular Ca^2+^. Representative Western blots showing the expression level of phosphorylated and total protein of Akt (A) or ERK1/2 (B). All conditions were stimulated by thrombin (100 nmol/L) for 15 min in Ca^2+^‐containing media, Ca^2+^‐free media, and in Ca^2+^‐free media in the presence of 10 *μ*mol/L CPA. Blots are representative of 3 independent sets of experiments.

We examined whether thrombin‐mediated Akt phosphorylation is dependent on extracellular and intracellular Ca^2+^ (Fig. 2). EGTA (2 mmol/L) was added to the extracellular solution to chelate extracellular Ca^2+^ for 15 min. In this EGTA‐containing media the phosphorylation of Akt was completely prevented (Fig. 2A). Likewise, pretreatment of the cells with CPA (10 *μ*mol/L) in the EGTA‐containing media to deplete the ER and SR calcium stores also prevented the phosphorylation of Akt. The same conditions had no significant effect upon the phosphorylation of ERK, another kinase, in the same cells (Fig. 2B), indicating a specificity of this effect on Akt. Thrombin‐induced Akt phosphorylation, therefore, requires the presence of extracellular and intracellular Ca^2+^.

### Long‐term serum starvation enhances thrombin‐mediated Akt phosphorylation in PASMC

**Figure 3.**
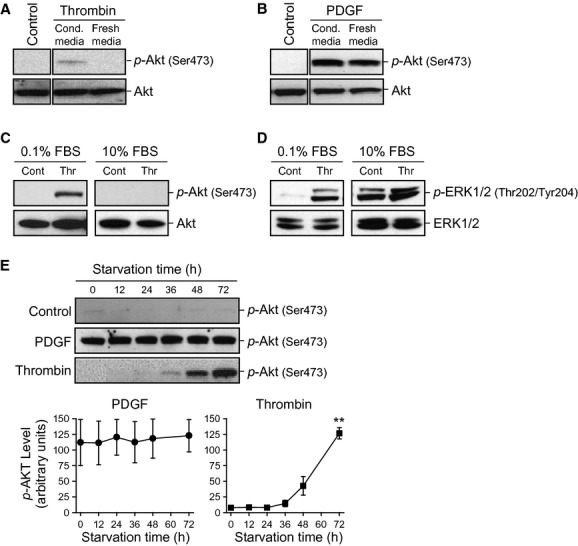
Serum deprivation of PASMC for >24 h enables thrombin‐dependent phosphorylation of Akt. PASMC were serum deprived for 48 h, thrombin (A) or PDGF (B) was then prepared with the conditioned (cond.) media that the cells had been grown in or with fresh media and added to the cells. Western blotting for total and phospho‐Akt was then performed. Representative Western blots show thrombin‐mediated phosphorylation and total protein of Akt (C) and ERK (D) in either serum‐deprived media (0.1% FBS) or in serum‐containing cell growth media (10% FBS). (E) shows the time dependence of the phosphorylation of Akt in serum‐starved cells with PDGF‐ or thrombin‐ treated for 15 min after serum starvation. Time course of p‐Akt in PDGF‐ and thrombin‐treated groups shown in arbitrary units. All blots were representative of 3 independent sets of experiments. **P <**0.01 versus 0 h.

Upon chelation of Ca^2+^ in the media (Fig. 2), we observed only partial recovery of Akt phosphorylation could be achieved after replacing the calcium‐free media. We examined the effects of conditioned media versus fresh media on thrombin and PDGF‐induced Akt phosphorylation (Fig. 3A and B). Cells were grown in smooth muscle cell growth media and serum‐starved for 48 h prior to either thrombin or PDGF stimulation. The effects of thrombin and PDGF on Akt phosphorylation in conditioned growth media was compared to fresh growth media. Thrombin‐induced Akt phosphorylation was only seen when prepared in conditioned media as opposed to fresh media, while PDGF was able to stimulate Akt phosphorylation in both conditioned and fresh media. As shown in Fig. 3C, thrombin had no significant effect on Akt phosphorylation in the presence of 10% fetal bovine serum, while in serum deprived conditions (0.1% FBS), there was an increase in Akt phosphorylation after thrombin stimulation. Interestingly serum deprivation was not required for thrombin‐induced ERK phosphorylation, though a greater increase in thrombin‐induced ERK phosphorylation was seen with 0.1% FBS conditions (Fig. 3D). We next assessed the time course of serum deprivation that was required for thrombin‐induced Akt phosphorylation. As shown in Fig. 3E, serum starvation without thrombin stimulation was not associated with Akt phosphorylation. Thrombin and PDGF (as a positive control) were added for a period of 15 min subsequent to serum starvation for the indicated time periods. Thrombin‐induced Akt phosphorylation is shown at 36 h of serum deprivation and further deprivation leads to increased Akt phosphorylation through 72 h. PDGF, on the other hand, was able to significantly induce the phosphorylation of Akt at each time point with no significant differences in phosphorylation up to 72 h.

### Thrombin induces Ca^2+^ entry and enhances SOCE in PASMC

**Figure 4.**
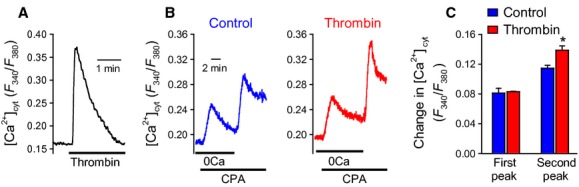
Thrombin induces a rise in [Ca^2+^]_cyt_ and enhances SOCE in PASMC. Representative traces showing changes in [Ca^2+^]_cyt_ in response to thrombin (5 nmol/L) in PASMC (A). Store‐operated calcium entry was assessed in PASMC (B) by passive depletion of ER stores using CPA under low Ca^2+^ conditions followed by restoration of normal extracellular Ca^2+^ (1.8 mmol/L). (C) Bar chart demonstrating enhanced SOCE after pretreatment with thrombin (100 nmol/L) was compared to vehicle. *P <**0.05.

Thrombin causes a transient rise of [Ca^2+^]_cyt_ in PASMC (Fig. 4A). Store‐operated calcium entry (SOCE) was elicited by passive ER calcium store depletion with CPA (10 *μ*mol/L), while maintaining low extracellular [Ca^2+^] (first peak). Upon repletion of extracellular [Ca^2+^] (1.8 mmol/L), there is an increase in intracellular [Ca^2+^] (second peak) through store‐operated calcium channels (SOCC). Thrombin (100 nmol/L) pretreatment for 24 h in human PASMC resulted in enhanced SOCE compared to untreated cells (Fig. 4B).

### Thrombin treatment increases proliferation in PASMC from both IPAH and CTEPH patients

**Figure 5.**
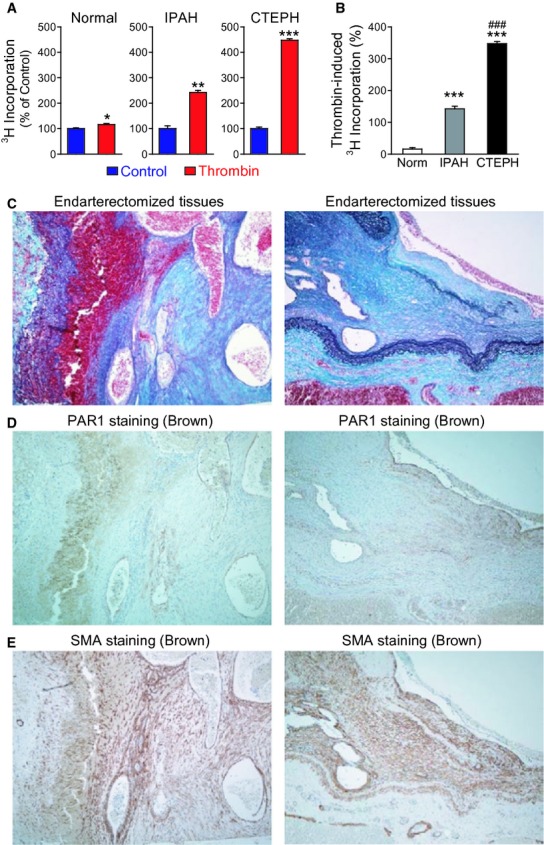
Thrombin‐induced proliferation in normal human PASMC, cells from patients with IPAH, and CTEPH. (A) Bar charts examining the effect of thrombin (100 nmol/L) in normal PASMC, IPAH PASMC, and CTEPH PASMC using ^3^H incorporation assay (in counts per minute, displayed as percentage vs. control). *P <**0.05, **P <**0.01, and ***P <**0.001 versus control (serum‐starved cells). (B) Bar chart comparing the ratio of thrombin‐induced proliferation in Normal, IPAH and CTEPH PASMC. ***P <**0.001 versus Normal PASMC; ^###^*P *<**0.001 versus IPAH. (C) Representative immunostaining showing Elastica‐Masson staining. (D) Representative immunostaining showing PAR1 staining (brown) localized to pulmonary artery endothelium and organized thrombus tissue. (E) Representative immunostaining showing smooth muscle actin staining (brown).

In cell proliferation assays, cells were serum‐starved for 48 h and the effect of thrombin (100 nmol/L) was assessed over a 24‐h time period (Fig. 5). In normal PASMC, thrombin did induce a small but significant increase in proliferation, while PASMC derived from patients with IPAH and CTEPH had significantly higher proliferation responses to thrombin (Fig. 5A). Interestingly, thrombin‐induced proliferation was significantly greater in CTEPH cells than both IPAH and normal PASMC (Fig. 5B).

### PAR1 is present in CTEPH tissue

In lung tissues of CTEPH, Elastica‐Masson staining shows layers of organized thrombi and multiple recanalized channels in the thrombi (Fig. 5C). PAR1, a thrombin receptor, staining was positive not only in endothelial cell layer but also in the cells in organized thrombi (Fig. 5D). PAR1 positive cells in the organized thrombi were also positive for smooth muscle actin staining (Fig. 5E).

### Thrombin treatment is associated with transient Akt and mTOR phosphorylation in CTEPH PASMC

**Figure 6.**
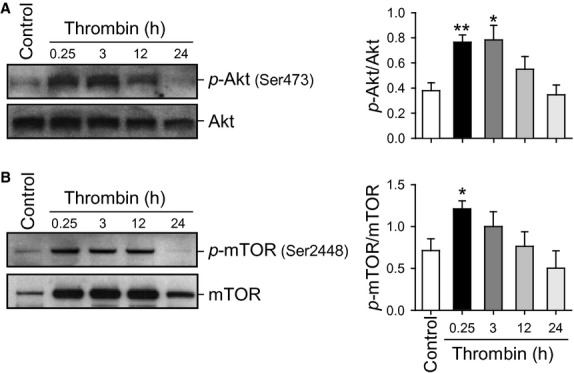
Time course of thrombin‐induced Akt/mTOR pathway phosphorylation in CTEPH PASMC. (A) Representative Western blots showing time‐dependent changes in phosphorylated and total proteins of Akt (A) and mTOR (B) induced by thrombin stimulation in CTEPH cells. Protein expression was assessed at 0.25, 3, 12, and 24 h after thrombin treatment. Summary data (mean ± SE, *n* = 3) were quantitated and compared at each time point with the control being without thrombin treatment. *P <**0.05 and **P <**0.01 versus Control (Cont) bars.

Consistent with the previous experiments in normal PASMC, CTEPH PASMC treated with thrombin transiently increased Akt and subsequently mTOR phosphorylation (Fig. 6). Cells were serum‐starved for 48 h and the effect of thrombin (100 mmol/L) was assessed over a 24 h time period.

**Figure 7.**
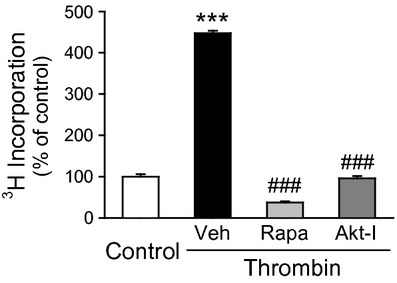
Thrombin‐induced proliferation is attenuated by inhibition of the Akt/mTOR pathway in CTEPH PASMC. Bar charts examine the effect of rapamycin (10 nmol/L) and Akt inhibitor (1 *μ*mol/L) on the thrombin‐ stimulated proliferation of CTEPH PASMC using ^3^H incorporation (in counts per minute, cpm). ***P <**0.001 versus control; ^###^*P *<**0.001 vs Veh.

### Thrombin‐induced proliferation is attenuated by both Akt and mTOR inhibition in CTEPH PASMC

As described previously, thrombin treatment is associated with increased thymidine uptake in CTEPH PASMC, indicating increased DNA synthesis and proliferation. To determine the contribution of Akt signaling to this effect, we used inhibitors of both Akt and mTOR (Fig. 7). A commercially available Akt inhibitor previously validated as a selective Akt 1/2 inhibitor (Calleja et al. 2009) was able to attenuate thymidine uptake. Rapamycin, which is known to have a primary effect of mTOR inhibition (Brown et al. 1994; Edinger et al. 2003), was also able to attenuate thymidine uptake in CTEPH PASMC. These findings indicate that each of these agents inhibit the proliferative effects of thrombin on CTEPH PASMC.

## Discussion

The current results highlight the potentially important role that thrombin plays on Akt phosphorylation in PASMC and the implications of this mechanism in disease pathogenesis of IPAH and CTEPH. We have implicated Akt phosphorylation in the effects of thrombin on both normal PASMC and CTEPH cells with transient increased Akt phosphorylation peaking around 3 h. Intracellular calcium regulation is shown to be an important mediator of the effects of thrombin as Akt phosphorylation can be blocked by chelation of extracellular [Ca^2+^] in the growth media or passive depletion of intracellular [Ca^2+^] by CPA, yet there is no similar effect on ERK phosphorylation, a parallel kinase pathway. Serum starvation appears to be an important mediator of thrombin's activity on Akt phosphorylation with the greatest effects seen after 72 h of serum starvation, an effect that is distinct from other similar pathways such as PDGF. Consistent with previous data (Wu et al. 2003; Firth et al. 2009), thrombin is also associated with a transient rise in intracellular calcium in PASMC, our current results show that this rise is at least in part due to enhanced SOCE.

As a part of the common coagulation cascade, thrombin plays an important role in thrombosis, an essential event in the development of CTEPH. Furthermore, thrombin is known to exert direct effects on intracellular signaling pathways in the pulmonary vasculature, which has previously been demonstrated to contribute to vascular injury (McNamara et al. 1993; Ghigliotti et al. 1998; Schror et al. 2010). Thrombin has been shown to mediate endothelial cell contraction and increased cell gap formation leading to direct PASMC exposure to thrombin (Vogel et al. 2000). Intracellular effects of thrombin are mediated through cleavage of the extracellular domain of protease‐activated receptors (PARs); G‐protein coupled receptors at the cell membrane (Vu et al. 1991; Coughlin 2000). PARs have been shown to impact downstream effects by phosphorylation of ERK and Akt in platelets and various vascular SMC (Madamanchi et al. 2001; Hunter et al. 2009; Smyth et al. 2009; Chung et al. 2010), yet limited data exist in PASMC (Gorlach et al. 2005). Here, we have shown the presence of PARs in CTEPH tissue and colocalization with SMαA staining that suggests smooth muscle cells, and smooth muscle progenitor cells, are presenting these receptors. We have shown that thrombin‐induced proliferation is greater in IPAH versus normal PASMC and that it is further increased in cells derived from CTEPH patients after pulmonary endarterectomy (PEA). Our previous study has shown this CTEPH tissue to contain a substantial presence of progenitor cells (Yao et al. 2009; Firth et al. 2010). We have identified smooth muscle alpha‐actin (SMαA) positive cells account for >85% of the total cell population, yet the diversity of staining density and pattern suggest an early, not fully differentiated smooth muscle cell line in the CTEPH thrombi. RT‐PCR results have confirmed early smooth muscle differentiation markers SMαA and transgelin along with intermediate filaments nestin and vimentin. Therefore, we have identified both similarities and differences between thrombin stimulation of normal PASMC, IPAH PASMC, and CTEPH PASMC in this study.

The intracellular signaling effects of thrombin have not previously been studied in CTEPH. Increased PASMC proliferation in and around CTEPH tissue will lead to vascular remodeling, a critical event leading to increased pulmonary vascular resistance and increased pulmonary arterial pressure (Moser and Bloor 1993). Similarly, IPAH is thought to confer a hypercoaguable state, with in situ thrombosis being a long‐known, yet poorly understood pathogenic characteristic of this disease (Pietra et al. 1989). Therefore, thrombin may likely play a pivotal role in IPAH as well as CTEPH and our data presented here implicate its importance in vascular remodeling.

A ubiquitous pathway in the human body, Akt/mTOR was first described in cancer research as an antiapoptotic mechanism (Vivanco and Sawyers 2002; Morgensztern and McLeod 2005). This pathway has since been shown to have multiple downstream effects in different cell types with cell survival shown to be an important global function (Datta et al. 1999; Mangi et al. 2003; Liu et al. 2008). Our current data suggest that thrombin mediates increased Akt phosphorylation in PASMC, which we propose will promote the pathogenesis of both CTEPH and IPAH through increased PASMC proliferation leading to medial hypertrophy and vascular remodeling. Recent study has shown the Akt/mTOR pathway to be important to PASMC proliferation in experimental animal models of PAH and hypoxia‐induced PASMC proliferation in vitro (Humar et al. 2002; Paddenberg et al. 2007; Agard et al. 2009; Houssaini et al. 2013). Our previous data suggest that this pathway plays an important role in store‐operated calcium entry and subsequently PASMC proliferation in endarterectomized tissue from CTEPH patients (Sacks et al. 2008; Ogawa et al. 2009). Our current data further provide evidence that the Akt/mTOR signaling pathway is necessary for thrombin‐mediated increases in proliferation in CTEPH PASMC.

This study examines the effects of thrombin on differing cell lines and under unique conditions. In PAEC, Akt phosphorylation has been associated with increased NO synthesis, a mechanism of vasorelaxation (Hisamoto et al. 2001; Morales‐Ruiz et al. 2001), yet our data that thrombin fails to lead to Akt phosphorylation in PAECs would lead us to believe that thrombin‐induced Akt phosophorylation is cell‐specific in the pulmonary vasculature leading to detrimental vascular remodeling rather than the protective effects that would result from NO synthesis. Calcium‐dependent phosphorylation is also shown to be specific to Akt in PASMC, with the parallel ERK pathway showing calcium‐independent phosphorylation. Previous study has shown both calcium‐independent and calcium‐dependent mechanisms of ERK and Akt phosphorylation (Shah and Catt 2002; Takahashi and Mendelsohn 2003; Schmitt et al. 2004; Li and Malik 2005; Xiang et al. 2010), and the current data suggest that these mechanisms are distinct among different stimuli and possibly in different cell lines. By understanding the predominant mechanism of injury, we can better target specific cell lines and kinase pathways either separately or synergistically.

The effects of thrombin on PASMC in this study appear to require serum deprivation for at least 24 h in order to phosphorylate Akt, a specific effect that was not seen with thrombin‐mediated ERK phosphorylation. We suggest that target receptor upregulation, cofactor secretion, or depletion of thrombin inhibitors may be involved in the requirement of serum deprivation for this effect. Previous study has also shown growth arrest‐specific gene *6* (Gas6) to be a cofactor that enhances thrombin‐induced proliferation in rat PASMC, yet lacks intrinsic activity alone (Nakano et al. 1995; Goruppi et al. 1996; Nagata et al. 1996). It will be interesting to evaluate if thrombin receptors, including PARs, are upregulated after serum deprivation or other secreted factors such as Gas6 may be working in conjunction with thrombin in order to mediate the phosphorylation of Akt. It must also be considered that an inhibitor of thrombin activity, such as antithrombin III, found in bovine serum is present that degrades over time.

We conclude that thrombin treatment induces cell proliferation and Akt phosphorylation in IPAH and CTEPH PASMC. We still are limited with our conclusions due to the fact that these CTEPH cells have not been clearly identified, yet appear to resemble immature SMC. [Ca^2+^]_cyt_ appears to be required for phosphorylation of Akt, yet the Akt/mTOR pathway also enhances the rise of [Ca^2+^]_cyt_ in PASMC through SOCE. Thrombin is known to be important in clotting and thrombus formation, but our data would implicate that thrombin may play a critical role in pathogenic vascular remodeling of both IPAH and CTEPH and may be a novel therapeutic target. Direct thrombin inhibitors have recently been used clinically as anticoagulants to treat acute venous thromboembolism and to prevent thrombosis in atrial fibrillation (Di Nisio et al. 2005; Schulman et al. 2009). The current data would suggest that there may be further effects that these medications have on intracellular signaling pathways in the pulmonary circulation. We hope to shed new light on these mechanisms in order to provide further evidence for use of these medications in both IPAH and CTEPH. We further show that Akt/mTOR is affected by and has important effects on [Ca^2+^]_cyt_ in PASMC, which has important implications in vascular remodeling of IPAH and CTEPH.

## Acknowledgments

This work was supported, in part, by grants from the National Heart, Lung, and Blood Institute of the National Institutes of Health (HL‐115014, HL‐066012, and HL‐098053). AO was supported by Japan Heart Foundation/Bayer Yakuhin Research Grant Abroad and ALF is supported by a Postdoctoral Training Fellowship from the California Institute of Regenerative Medicine.

## Conflict of Interest

None declared.
